# Causal Relationship Between Gut Microbiota and Autoimmune Diseases: A Two-Sample Mendelian Randomization Study

**DOI:** 10.3389/fimmu.2021.746998

**Published:** 2022-01-24

**Authors:** Qian Xu, Jing-Jing Ni, Bai-Xue Han, Shan-Shan Yan, Xin-Tong Wei, Gui-Juan Feng, Hong Zhang, Lei Zhang, Bin Li, Yu-Fang Pei

**Affiliations:** ^1^ Department of Epidemiology and Biostatistics, School of Public Health, Medical College of Soochow University, Suzhou, China; ^2^ Jiangsu Key Laboratory of Preventive and Translational Medicine for Geriatric Diseases, Medical College of Soochow University, Suzhou, China; ^3^ Center for Genetic Epidemiology and Genomics, School of Public Health, Medical College of Soochow University, Suzhou, China; ^4^ Department of General Surgery, Suzhou Ninth Hospital Affiliated to Soochow University, Affiliated Wujiang Hospital of Nantong University, Suzhou, China

**Keywords:** Mendelian randomization, gut microbiota, autoimmune disease (AD), type 1 diabetes, celiac disease

## Abstract

**Background:**

Growing evidence has shown that alterations in gut microbiota composition are associated with multiple autoimmune diseases (ADs). However, it is unclear whether these associations reflect a causal relationship.

**Objective:**

To reveal the causal association between gut microbiota and AD, we conducted a two-sample Mendelian randomization (MR) analysis.

**Materials and Methods:**

We assessed genome-wide association study (GWAS) summary statistics for gut microbiota and six common ADs, namely, systemic lupus erythematosus, rheumatoid arthritis, inflammatory bowel disease, multiple sclerosis, type 1 diabetes (T1D), and celiac disease (CeD), from published GWASs. Two-sample MR analyses were first performed to identify causal bacterial taxa for ADs in discovery samples. Significant bacterial taxa were further replicated in independent replication outcome samples. A series of sensitivity analyses was performed to validate the robustness of the results. Finally, a reverse MR analysis was performed to evaluate the possibility of reverse causation.

**Results:**

Combining the results from the discovery and replication stages, we identified one causal bacterial genus, *Bifidobacterium*. A higher relative abundance of the *Bifidobacterium* genus was associated with a higher risk of T1D [odds ratio (OR): 1.605; 95% CI, 1.339–1.922; *P_FDR_* = 4.19 × 10^−7^] and CeD (OR: 1.401; 95% CI, 1.139–1.722; *P_FDR_* = 2.03 × 10^−3^), respectively. Further sensitivity analyses validated the robustness of the above associations. The results of reverse MR analysis showed no evidence of reverse causality from T1D and CeD to the *Bifidobacterium* genus.

**Conclusion:**

This study implied a causal relationship between the *Bifidobacterium* genus and T1D and CeD, thus providing novel insights into the gut microbiota-mediated development mechanism of ADs.

## Introduction

Autoimmune diseases (ADs) are conditions in which an individual’s immune system mistakenly attacks its host’s tissues. Patients with ADs often endure lifelong debilitating symptoms, loss of organ function, reduced productivity at work, and high medical expenses. ADs are considered a significant cause of morbidity and mortality worldwide. Accumulating evidence demonstrates a steady rise in the incidence of ADs over the last few decades (1).

Although the etiology and pathogenesis of ADs are not fully understood, genetic components, environmental factors, and their interactions have great significance in their development. In addition, growing evidence suggests that alterations in gut microbiota composition are closely related to autoimmunity (2, 3). The gut microbiota is defined as the community of microorganisms that live in the human gastrointestinal tract. Gut microbial dysbiosis has been observed in many AD studies. For example, multiple studies reported a decrease of *Firmicutes*/*Bacteroidetes* ratio in systemic lupus erythematosus (SLE) patients and type 1 diabetes (T1D) patients (4, 5). A case-control study reported an increased abundance of *Methanobrevibacter* and *Akkermansia* and decreased abundance of *Butyricimonas* in patients with multiple sclerosis (MS) (6). Chen et al. (7) found that rheumatoid arthritis (RA) patients had a decrease in *Faecalibacterium* and expansion of *Eggerthella* and *Collinsella*.

All the above gut microbiota–AD associations were derived from cross-sectional studies, leaving the causal nature of these associations elusive. However, establishing causal relationships not only deepens the understanding of gut microbiota-derived AD pathogenesis but also has the capacity to guide microbiota-orientated interventions against AD in the clinic. Therefore, there is an urgent need to elucidate the causal relationship between the gut microbiota and various types of AD.

Mendelian randomization (MR) is a statistical approach that implies causal association from an exposure to an outcome. It uses genetic variants associated with exposure as a surrogate for exposure to assess the association between the surrogate and the outcome (8). Thanks to fruitful findings from large-scale genome-wide association studies (GWASs) conducted to date at both gut microbiota and disease levels (9–11), MR analysis has been widely applied to various scenarios, including the causal associations between gut microbiota and AD. In previous studies, García-Santisteban et al. (12) performed an MR analysis and identified a causal association between gut microbiota composition and celiac disease (CeD). Another study by Inamo (13) identified no causal association between gut microbiota composition and RA. The above two studies fall short in that they treat gut microbiota composition as a whole without distinguishing specific taxa, while different microbial taxa may have distinct effects on human health. During the preparation of this article, Zhang et al. (14) and Xiang et al. (15) investigated the causal effects of specific microbial taxa on two ADs, inflammatory bowel disease (IBD) and SLE. However, studies on other ADs are still sparse.

In the present study, aiming to investigate the causal relationship between gut microbiota and a broad range of ADs, we conducted a comprehensive two-sample MR analysis of six ADs, including SLE, RA, IBD, MS, T1D, and CeD.

## Materials and Methods

### Ethics Statement

**Figure 1:**
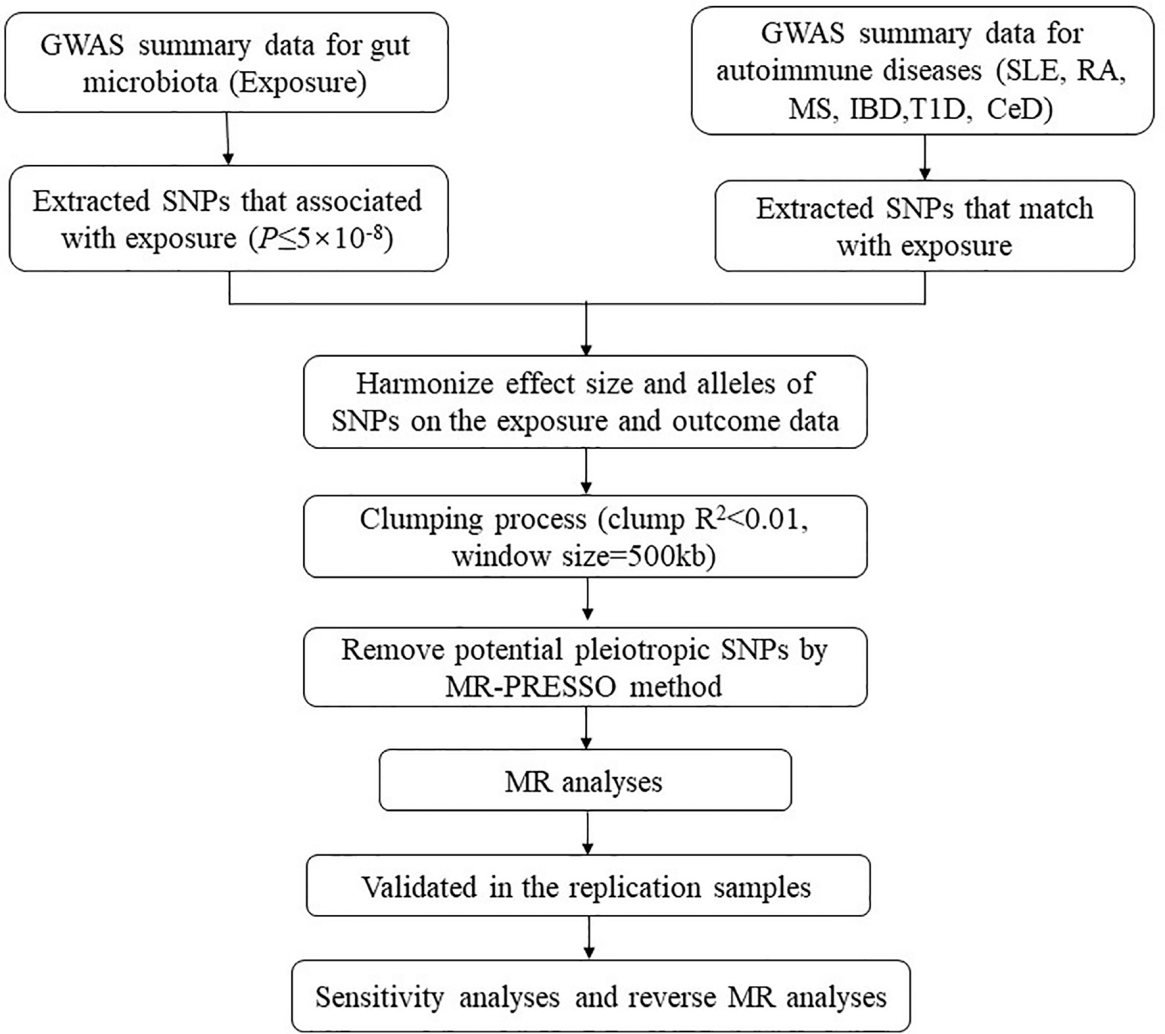
The flowchart of the study. The whole workflow of MR analysis. MR, Mendelian randomization; SLE, systemic lupus erythematosus; RA, rheumatoid arthritis; MS, multiple sclerosis; IBD, inflammatory bowel disease; T1D, type 1 diabetes; CeD, celiac disease.

Our analysis used publicly available GWAS summary statistics. No new data were collected, and no new ethical approval was required. The flowchart of the study is shown in **Figure 1**. Briefly, gut microbiota served as the exposure, while ADs served as the outcome. Single-nucleotide polymorphisms (SNPs) significantly associated with specific gut microbiota taxa were selected as instrumental variables (IVs) based on strict inclusion and exclusion criteria. Outcome samples included both discovery and replication samples. A series of sensitivity analyses was performed for significant associations. Finally, reverse MR analysis was performed to mitigate the potential impact of ADs on the causal gut microbiota.

### Gut Microbiota Sample

Summary statistics for gut microbial taxa were obtained from a large-scale multi-ethnic GWAS meta-analysis that included 18,340 individuals from 24 cohorts (16). The microbial composition was profiled by targeting three distinct variable regions of the 16S rRNA gene. To account for differences in sequencing depth, all datasets were rarefied to 10,000 reads per sample. Taxonomic classification was performed using direct taxonomic binning. In each cohort, only the taxa present in more than 10% of the samples were included to explore the effect of host genetics on the abundance of gut bacterial taxa. The study-wide cutoffs included an effective sample size of at least 3,000 individuals and presence in at least three cohorts. A total of 211 taxa (131 genera, 35 families, 20 orders, 16 classes, and 9 phyla) were included. After adjustment for age, sex, technical covariates, and genetic principal components, Spearman’s correlation analysis was performed to identify genetic loci that affected the covariate-adjusted abundance of bacterial taxa. More details on the microbiota data were described elsewhere (16).

### Autoimmune Disease Discovery Samples

In the discovery stage, GWAS summary statistics for each of the six ADs were extracted from publicly available GWAS analyses. Summary statistics for SLE were obtained from a publicly available GWAS meta-analysis, including 7,219 cases and 15,991 controls of European ancestry (17). Summary statistics for RA were extracted from a GWAS meta-analysis, including 14,361 RA cases and 43,923 controls of European ancestry from 18 studies (18). Summary statistics for IBD were obtained from a GWAS meta-analysis of 25,042 IBD cases and 34,915 controls of European ancestry (19). Summary statistics of MS were derived from the discovery stage of the latest GWAS meta-analysis of the International MS Genetics Consortium (IMSGC), including 14,802 MS cases and 26,703 controls of European ancestry (20). Summary statistics of T1D were derived from a GWAS with 6,683 T1D cases and 12,173 controls of European ancestry (21). Finally, summary statistics of CeD were obtained from a GWAS meta-analysis, including 12,041 CeD cases and 12,228 controls (22). Detailed information on the datasets is provided in **Table 1**.

**Table 1:** Autoimmune diseases GWAS samples used in this study.

Stage	Trait	N. cases	N. controls	Populations	Reference
Discovery	SLE	7,219	15,991	European	Bentham et al. (17)
	RA	14,361	43,923	European	Okada et al. (18)
	IBD	25,042	34,195	European	de Lange et al. (19)
	MS	14,802	26,703	European	Patsopoulos et al. (20)
	T1D	6,683	12,173	European	Onengut-Gumuscu et al. (21)
	CeD	12,041	12,228	97% European	Trynka et al. (22)
Replication	SLE	907	1,524	Spain	Julià et al. (23)
	RA	5,082	447,182	British	UKBB (data filed: 20002)
	IBD	3,878	448,386	British	UKBB (data filed: 20002)
	MS	1,406	450,858	British	UKBB (data filed: 41202 41204)
	T1D	3,041	449,223	British	UKBB (data filed: 41202 41204)
	CeD	4,533	10,750	European	Dubois et al. (24)

SLE, systemic lupus erythematosus; RA, rheumatoid arthritis; MS, multiple sclerosis; IBD, inflammatory bowel disease; T1D, type 1 diabetes; CeD, celiac disease; GWAS, genome-wide association study; UKBB, UK Biobank.

### Autoimmune Disease Replication Samples

Significant bacterial taxa identified in the discovery stage were replicated during the replication stage. The replication outcome samples for RA, IBD, MS, and T1D were obtained from the UK Biobank study, which is a large prospective cohort study with approximately 500,000 participants aged 40–69 years from 22 centers across the United Kingdom. The replication sample of SLE was a single GWAS from Spain, including 907 patients with SLE and 1,524 healthy controls (23). The replication sample for CeD is a GWAS meta-analysis of five samples, including 4,533 CeD cases and 10,750 controls of European ancestry (24). Detailed information on the replication samples is presented in **Table 1**.

### Selection of Instrumental Variables

The 211 bacterial taxa were categorized at six taxonomic levels. Of these, the genus is the smallest and most specific taxonomic level. To identify each causal bacteria group as specifically as possible, we analyzed 131 bacterial taxa at the genus level only. Fourteen taxa with unknown groups were excluded, meaning 117 bacterial taxa were included in the subsequent MR analysis.

SNPs associated with gut bacterial taxa at the genome-wide significance threshold *P* < 5.0 × 10^−8^ were selected as potential IVs. A series of quality control steps was implemented to select eligible IVs. First, SNPs with inconsistent alleles between the exposure and outcome samples (i.e., A/G vs. A/C) were excluded. Second, palindromic A/T or G/C alleles were excluded. Third, SNPs within each bacterial taxon were clumped to retain only independent SNPs. The linkage disequilibrium (LD) threshold for clumping was set to r^2^ < 0.01, and the clumping window size was set to 500 kb. LD was estimated based on the European-based 1,000 Genome Projects reference panel. Fourth, the MR pleiotropy residual sum and outlier (MR‐PRESSO) test was applied to detect potential horizontal pleiotropy and to eliminate the effects of pleiotropy by removing outliers (25). Finally, to assess the strength of the selected SNPs, the following equation was used to calculate the *F* statistics for each bacterial taxon:


$$F=\frac{R^{2}(n-1-k)}{(1-R^{2})k}$$

where *R* ^2^ is the portion of exposure variance explained by the IVs, *n* is the sample size, and *k* is the number of IVs. An *F*-statistic ≥10 indicates no strong evidence of weak instrument bias (26). IVs with *F*-statistics less than <10 were considered weak IVs and were excluded.

### Statistical Analysis

We performed MR analysis to estimate the causal effect of the gut microbiota on the six ADs. For bacterial genera containing only one SNP, the Wald ratio method was used for the MR analysis. The causal effect was calculated by dividing the SNP-outcome effect estimated by the SNP-exposure effect estimate. For bacterial genera containing multiple SNPs, multiple tests, including fixed-/random-effects inverse variance weighted (IVW) test (27), weighted median method, and MR-Egger regression test were performed. Cochrane’s *Q* test was performed to assess the heterogeneity among SNPs associated with each bacterial genus. In the presence of heterogeneity (*P* < 0.05), the random-effects IVW test was used instead to provide a more conservative but robust estimate. The weighted median test can generate consistent estimates when ≥50% of the weights come from valid IVs (28). The MR-Egger regression test allows pleiotropy present in more than 50% of IVs (29).

Significant genera identified in the discovery samples were replicated in replication samples. The replication MR analysis procedure was the same as that used in the discovery stage. To evaluate the robustness of the identified causal associations, we performed two sensitivity analyses, including the MR-Egger intercept test and leave-one-out analysis. The intercept of the MR-Egger regression test can provide an estimate of the degree of directional pleiotropy (29). The leave-one-out analysis was performed to evaluate whether the significant results were driven by a single SNP.

### Reverse Mendelian Randomization Analysis

To explore whether ADs have any causal impact on the identified significant bacterial genus, we also performed a reverse MR analysis (i.e., ADs as exposure and the identified causal bacterial genus as outcome) using SNPs that are associated with ADs as IVs.

All statistical analyses were conducted using R (version 4.0.3). The IVW, weighted median, and MR-Egger regression methods were performed using the “*TwoSampleMR*” package (version 0.5.4). The MR-PRESSO test was performed using the “*MRPRESSO*” package. The statistical significance of the MR effect estimates was defined as a false discovery rate (FDR) of <5% to adjust for multiple testing.

## Results

### Selection of Instrumental Variables

After a series of quality control steps, 32 SNPs associated with 13 genera were selected as IVs. Specifically, 19 independent SNPs (*P* < 5.0 × 10^−8^, r^2^ < 0.01) were associated with 13 genera for SLE, 17 independent SNPs were associated with 12 genera for RA, 19 SNPs were associated with 13 genera for MS, 18 SNPs were associated with 12 genera for IBD, 7 SNPs were associated with 3 genera for T1D, and 6 SNPs were associated with 3 genera for CeD (**Supplementary Table S1**). No evidence of pleiotropic effects was detected by the MR-PRESSO global test (*P* > 0.05). The *F-*statistics of IVs ranged between 29.78 and 2,074.13, all largely >10, indicating no evidence of weak instrument bias (**Supplementary Table S2**).

### Causal Effects of Gut Microbiota on Autoimmune Diseases

In the discovery stage, the genetically predicted relative abundance of two genera, *Bifidobacterium* and *Ruminococcus*, was associated with the risk of SLE, MS, T1D, and CeD. *Ruminococcus* was also associated with the risk of IBD (**Table 2**). Specifically, a higher genetically predicted *Bifidobacterium* level was associated with a lower risk of SLE [odds ratio (OR): 0.565, 95% confidence interval (CI): 0.426–0.748, *P_FDR_* = 8.53 × 10^−4^]. In contrast, a higher genetically predicted *Bifidobacterium* was associated with a higher risk of MS (OR: 1.384, 95% CI: 1.128–1.700, *P_FDR_* = 0.012), T1D (OR: 1.605, 95% CI: 1.339–1.922, *P_FDR_* = 4.19 × 10^−7^), and CeD (OR: 1.401, 95% CI: 1.139–1.722, *P_FDR_* = 2.03 × 10^−3^). These associations were also supported by the weighted median method, as shown in **Table 2**. The genetically predicted *Ruminococcus* level was associated with a higher risk of SLE (OR: 5.593, 95% CI: 2.079–15.045, *P_FDR_* = 4.22 × 10^−3^), IBD (OR: 2.141, 95% CI: 1.425–3.216, *P_FDR_* = 2.92 × 10^−3^), and MS (OR: 2.890, 95% CI: 1.669–5.003, *P_FDR_* = 1.96 × 10^−3^). But its associations with T1D and CeD were negative (OR: 0.122, 95% CI: 0.0661–0.224, *P_FDR_* = 3.38 × 10^−11^) and CeD (OR: 0.352, 95% CI: 0.195–0.635, *P_FDR_* = 1.57 × 10^−3^). As shown in **Supplementary Table S2**, there was no evidence of a causal association between any microbial taxa and RA.

**Table 2:** Significant MR analysis results in the discovery samples.

Traits (outcome)	Bacterial taxa (exposure)	MR method	No. SNP	*F*-statistics	OR	95% CI	*P*	*P_FDR_*
SLE	*Bifidobacterium*	IVW (fixed)	6	2074.13	0.565	0.426–0.748	6.56 × 10^-5^	**8.53 × 10^-4^**
		Weighted median			0.508	0.353–0.730	2.50 × 10^-4^	**3.35 × 10^-3^**
		MR-Egger			0.776	0.132–4.538	0.792	0.819
	*Ruminococcus*	Wald ratio	1	31.33	5.593	2.079–15.045	6.50 × 10^-4^	**4.22 × 10^-3^**
IBD	*Bifidobacterium*	IVW (fixed)	6	1905.96	1.182	1.039–1.345	0.011	0.064
		Weighted median			1.182	1.009–1.384	0.037	0.188
		MR-Egger			1.226	0.641–2.344	0.561	0.767
	*Ruminococcus*	Wald ratio	1	31.33	2.141	1.425–3.216	2.43 × 10^-4^	**2.92 × 10^-3^**
MS	*Bifidobacterium*	IVW (fixed)	6	2074.13	1.384	1.128–1.698	1.84 × 10^-3^	**0.012**
		Weighted median			1.439	1.104–1.877	7.19 × 10^-3^	**0.047**
		MR-Egger			1.024	0.348–3.011	0.967	0.970
	*Ruminococcus*	Wald ratio	1	31.33	2.890	1.669–5.003	1.51 × 10^-4^	**1.96 × 10^-3^**
T1D	*Bifidobacterium*	IVW (fixed)	5	1804.95	1.605	1.339–1.922	2.79 × 10^-7^	**4.19 × 10^-7^**
		Weighted median			1.745	1.405–2.167	4.66 × 10^-7^	**6.99 × 10^-7^**
		MR-Egger			3.046	0.580–15.992	0.279	0.419
	*Ruminococcus*	Wald ratio	1	27.47	0.122	0.0661–0.224	1.13 × 10^-11^	**3.38 × 10^-11^**
CeD	*Bifidobacterium*	IVW (fixed)	4	981.22	1.401	1.139–1.722	1.35 × 10^-3^	**2.03 × 10^-3^**
		Weighted median			1.463	1.149–1.863	1.96 × 10^-3^	**2.95 × 10^-3^**
		MR-Egger			2.079	0.646–6.680	0.344	0.516
	*Ruminococcus*	Wald ratio	1	31.32	0.352	0.195–0.635	5.25 × 10^-4^	**1.57 × 10^-3^**

No. SNP is the number of SNPs being used as IVs.

Significant P_FDR_ was marked in bold.

MR, Mendelian randomization; SNP, single-nucleotide polymorphism; IVW, inverse-variance weighted; OR, odds ratio; CI, confidence interval; P_FDR_, P value corrected by false discovery rate (FDR); SLE, systemic lupus erythematosus; IBD, inflammatory bowel disease; MS, multiple sclerosis; T1D, type 1 diabetes; CeD, celiac disease.

These two genera *Bifidobacterium* and *Ruminococcus* were replicated in the replication samples. The causal effects of the *Bifidobacterium* genus on T1D and CeD were successfully replicated, as shown in **Table 3**. The effect direction was consistent with that in the discovery sample, which strengthened the confidence of the true causal associations.

**Table 3:** Results of the identified bacterial taxa in the replication samples.

Traits (outcome)	Bacterial taxa (exposure)	MR methods	No. SNP	*F*-statistics	OR	95% CI	*P*	*P_FDR_*
SLE	*Bifidobacterium*	IVW (fixed)	6	2,074.13	1.269	0.929–1.734	0.155	0.155
		Weighted median			1.175	0.801–1.723	0.410	0.410
		MR-Egger			0.849	0.124–5.808	0.876	0.876
	*Ruminococcus*	Wald ratio test	1	31.33	4.314	0.576–32.286	0.155	0.309
IBD	*Bifidobacterium*	IVW (fixed)	5	1,557.97	0.998	0.998–1.002	0.761	0.111
		Weighted median			0.998	0.995–1.001	0.142	0.142
		MR-Egger			1.002	0.987–1.016	0.845	0.850
	*Ruminococcus*	Wald ratio test	1	31.33	0.995	0.989–1.001	0.111	0.142
MS	*Bifidobacterium*	IVW (fixed)	5	1,557.97	1.001	0.999–1.003	0.070	0.140
		Weighted median			1.001	1.000–1.003	0.038	0.076
		MR-Egger			1.000	0.993–1.008	0.938	0.938
	*Ruminococcus*	Wald ratio test	1	31.33	1.002	0.998–1.006	0.312	0.312
T1D	*Bifidobacterium*	IVW (fixed)	5	1,557.97	1.002	1.001–1.004	8.58 × 10^-4^	**1.72 × 10^-3^**
		Weighted median			1.002	1.000–1.004	6.42 **×** 10^-3^	**0.013**
		MR-Egger			1.000	0.991–1.009	0.965	0.965
	*Ruminococcus*	Wald ratio test	1	31.33	0.997	0.991–1.002	0.247	0.247
CeD	*Bifidobacterium*	IVW (fixed)	6	2,207.82	1.643	1.300–2.076	3.16 × 10^-5^	**3.16 × 10^-5^**
		Weighted median			1.755	1.314–2.343	1.38 × 10^-4^	**1.38 × 10^-4^**
		MR-Egger			1.202	0.219–6.589	0.842	0.842

No. SNP is the number of SNPs being used as IVs.

Significant P_FDR_ was marked in bold.

MR, Mendelian randomization; SNP, single-nucleotide polymorphism; IV, instrumental variable; IVW, inverse-variance weighted; OR, odds ratio; CI, confidence interval; P_FDR_, P value corrected by false discovery rate (FDR); SLE, systemic lupus erythematosus; IBD, inflammatory bowel disease; MS, multiple sclerosis; T1D, type 1 diabetes; CeD, celiac disease.

### Sensitivity Analyses

**Figure 2:**
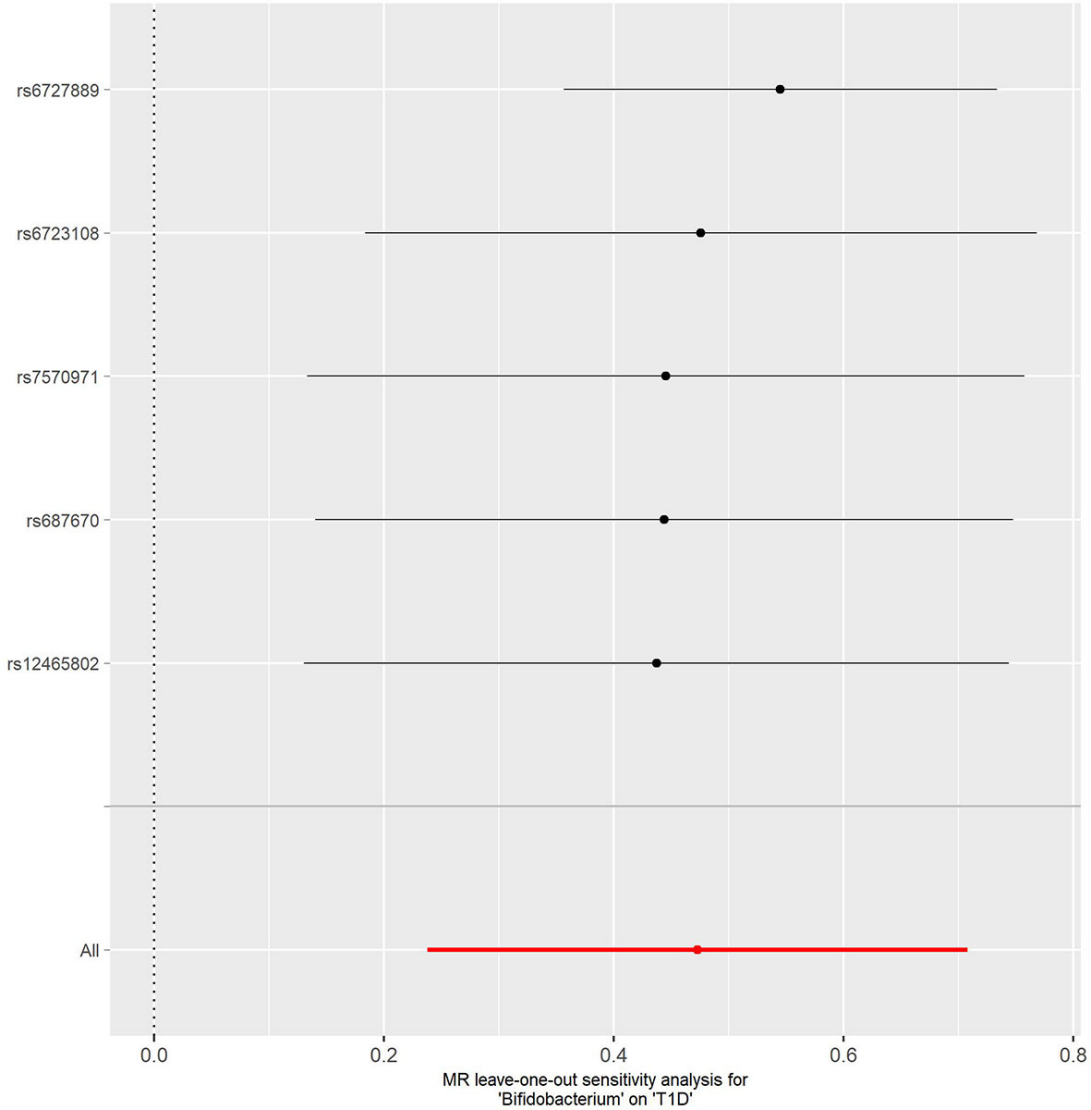
Leave-one-out analysis of the causal effect of *Bifidobacterium* on T1D. Red lines represent estimations from the IVW test. T1D, type 1 diabetes; IVW, inverse variance weighted.

**Figure 3:**
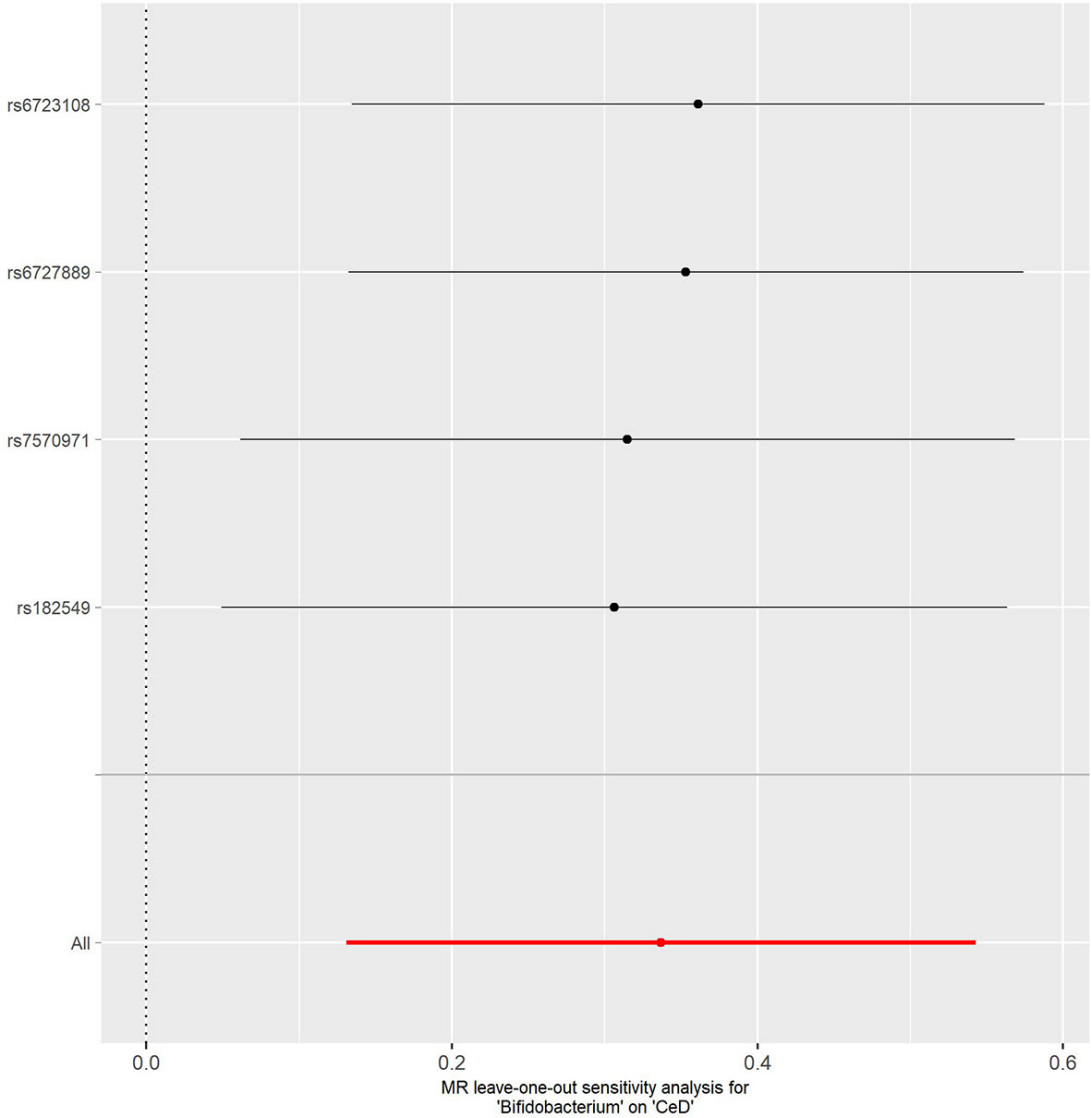
Leave-one-out analysis of the causal effect of *Bifidobacterium* on CeD. Red lines represent estimations from the IVW test. CeD, celiac disease; IVW, inverse variance weighted.

No evidence of heterogeneity was observed between the genetic IVs for *Bifidobacterium* (**Supplementary Table S3**). None of the MR-Egger regression intercepts deviated from null, indicating no evidence of horizontal pleiotropy (all intercept *P* > 0.05) (**Supplementary Table S4**). Additionally, the leave-one-out analysis showed no marked difference in causal estimations of *Bifidobacterium* on T1D and CeD, suggesting that none of the identified causal associations were driven by any single IV (**Figures 2**, **3**). In reverse MR analysis, there was no evidence of a causal effect of T1D and CeD on *Bifidobacterium* (**Table 4**). Detailed information on the IVs used in the reverse MR analyses is shown in **Supplementary Table S5**.

**Table 4:** Reverse causal association between ADs and gut microbiota.

Exposure	Outcome	MR method	No. SNP	OR	95% CI	*P*
T1D	*Bifidobacterium*	IVW	39	0.991	0.973–1.009	0.31
		Weighted median		0.993	0.964–1.023	0.66
		MR-Egger		0.990	0.995–1.026	0.58
CeD	*Bifidobacterium*	IVW	105	0.998	0.993–1.003	0.40
		Weighted median		0.998	0.990–1.005	0.56
		MR-Egger		0.993	0.986–1.000	0.08

No. SNP is the number of SNPs being used as IVs.

MR, Mendelian randomization; AD, autoimmune disease; SNP, single-nucleotide polymorphism; IV, instrumental variable; IVW, inverse-variance weighted; OR, odds ratio; CI, confidence interval; T1D, type 1 diabetes; CeD, celiac disease.

## Discussion

In this study, we performed two-sample MR analyses to investigate the causal association between gut microbiota and six common ADs (SLE, RA, MS, IBD, T1D, and CeD). Combining evidence from both discovery and replication samples, we identified that the bacterial genus *Bifidobacterium* was causally associated with the risk of T1D and CeD.

*Bifidobacterium* is the primary microbe that colonizes the human gut. Previous observational studies have demonstrated that *Bifidobacterium* plays an important role in the pathogenesis of multiple ADs. However, observational studies have yielded conflicting results regarding the effect pattern. Two case-control studies showed that the relative abundance of *Bifidobacterium* was higher in T1D patients than that in controls (30, 31). Similarly, a higher relative abundance of *Bifidobacterium* was observed in patients with CeD (32). In line with these studies, our study suggested that the increased relative abundance of *Bifidobacterium* was causally associated with a higher risk of T1D and CeD, indicating its harmful effect on both diseases. In contrast, several other studies observed a lower relative abundance of *Bifidobacterium* in T1D and CeD patients, suggesting its protective effect (33–35).

Recent studies have shown that probiotic intervention, mainly of the *Lactobacillus* and *Bifidobacterium* genera, can effectively attenuate the progression of multiple ADs, including T1D and CeD. In a double-blinded, placebo-controlled trial, probiotic intervention with *Bifidobacterium breve* BR03 and *B. breve* B632 has shown a positive effect on decreasing the production of the pro-inflammatory cytokine tumor necrosis factor-α (TNF-α) in children with CeD on a gluten-free diet (36). In contrast, Smecuol et al. (37) did not detect significant changes in TNF-α in CeD patients treated with *Bifidobacterium infantis*. Similarly, Groele et al. (38) reported that administration of *Lactobacillus rhamnosus* GG and *Bifidobacterium lactis* Bb12 had no significant effect on maintaining the residual pancreatic beta-cell function in children with newly diagnosed T1D. There was no significant difference in cytokine levels and intestinal permeability (zonulin levels) between the probiotics and placebo groups (38).

Some functional studies have shown evidence of the anti-inflammatory effects of *Bifidobacterium*, while others have reported its pro-inflammatory effects. A previous study showed that *Bifidobacterium adolescentis* significantly increased Th17 cell levels in several other gut-associated organs, while elevated Th17 cell responses have been associated with autoimmune/inflammatory disease in both mice and humans (38). In addition, López et al. (39) reported that some *Bifidobacterium bifidum* strains could induce the secretion of large amounts of interleukin IL-17 and promote Th17 cell polarization. Combining evidence from observational studies, MR analysis, clinical trials, and functional studies, we speculated that the positive and negative effects of *Bifidobacterium* on ADs may be species- and strain-specific. The causal relationship between *Bifidobacterium* and ADs needs to be further explored at more specialized levels (i.e., species level and strain level).

In previous studies, Zhang et al. (14) and Xiang et al. (15) performed MR analyses to investigate the effects of gut microbiota on IBD and SLE, respectively. Our study differs from their studies in the following three aspects: First, our study is more comprehensive in its investigation of ADs. Unlike the above two studies that analyzed two separate diseases, we comprehensively analyzed six common AD diseases. This will give us an opportunity to evaluate common gut microbiota that are causally related to multiple ADs. Second, the quality control procedure for selecting IVs was stricter in our study. We selected independent GWAS SNPs as IVs and conducted a series of sensitivity analyses, including horizontal pleiotropy assessment and reverse MR analysis, to maximally fulfill basic MR assumptions. In contrast, the above two studies used a fairly loose *P*-value threshold (*P* < 1 × 10^−5^) to select eligible IVs. Third, Zhang et al. (14) used summary-level data of gut microbiota in a relatively small sample size (N = 1,126 twin pairs). Instead, the sample size in the present study was much larger (N = 18,340). Meanwhile, the causal associations identified in the discovery stage were further replicated in independent replication outcome samples, which enhanced the confidence of the true causal relationship.

Nevertheless, our study had several limitations. First, while the majority of participants in the GWAS summary data used in our study were of European ancestry, a small number of the gut microbiota data were taken from sets consisting of other races, which may partially bias our estimates. Second, bacterial taxa were only analyzed at the genus level but not at a more specialized level such as species or strain levels. When microbiota GWASs use more advanced shotgun metagenomic sequencing analysis, the results can be more specific and accurate. Third, our study used gut microbiota data from a meta-analysis of mostly adult individuals, whereas the CeD study was conducted in children. Finally, most ADs are more prevalent in women than in men (e.g., SLE, RA, and MS). However, our study did not analyze the two genders separately, which may have influenced our results. It would be helpful to perform a gender-specific MR analysis in future endeavors.

In conclusion, our findings support the potentially causal effects of the *Bifidobacterium* genus on T1D and CeD. Although *Bifidobacterium* is generally considered beneficial bacteria, specific species and strains of *Bifidobacterium* may have varying effects on human health. Therefore, the potential mechanisms of specific species and strains of *Bifidobacterium* in the development of AD need to be further investigated.

## Data Availability Statement

The datasets presented in this study can be found in online repositories. The names of the repository/repositories and accession number(s) can be found in the article/**Supplementary Material**.

## Author Contributions

LZ and Y-FP designed the research. QX and J-JN collected the data. QX and BL analyzed the data. J-JN, B-XH, S-SY, X-TW, G-JF, and HZ performed the literature search. QX and BL drafted the article. Y-FP and LZ jointly supervised the study. All authors were involved in writing the paper. All authors contributed to the article and approved the submitted version.

## Funding

This study was partially supported by funding from the National Natural Science Foundation of China (31771417) and a project funded by the Priority Academic Program Development (PAPD) of Jiangsu higher education institutions. The numerical calculations in this paper have been done on the supercomputing system of the National Supercomputing Center in Changsha. This study was supported by grants from Suzhou Science and Technology Bureau (No. SKY2021022), Gusu Health Top-Notch Youth Talent of Suzhou Health Commission (No. GSWS2019086) and Wujiang District Health Commission (No. WWK201806).

## Conflict of Interest

The authors declare that the research was conducted in the absence of any commercial or financial relationships that could be construed as a potential conflict of interest.

## Publisher’s Note

All claims expressed in this article are solely those of the authors and do not necessarily represent those of their affiliated organizations, or those of the publisher, the editors and the reviewers. Any product that may be evaluated in this article, or claim that may be made by its manufacturer, is not guaranteed or endorsed by the publisher.
